# Characteristics of Carbapenem-Resistant and Colistin-Resistant *Escherichia coli* Co-Producing NDM-1 and MCR-1 from Pig Farms in China

**DOI:** 10.3390/microorganisms7110482

**Published:** 2019-10-23

**Authors:** Zhong Peng, Xiaosong Li, Zizhe Hu, Zugang Li, Yujin Lv, Minggang Lei, Bin Wu, Huanchun Chen, Xiangru Wang

**Affiliations:** 1 State Key Laboratory of Agricultural Microbiology, College of Animal Science and Veterinary Medicine, Huazhong Agricultural University, Wuhan 430040, China; pengzhong@ail.hzau.edu.cn (Z.P.); lixiaosong@webmail.hzau.edu.cn (X.L.); huzizhede@webmail.hzau.edu.cn (Z.H.); lizugang@webmail.hzau.edu.cn (Z.L.); wub@mail.hzau.edu.cn (B.W.); chenhch@mail.hzau.edu.cn (H.C.); 2 The Cooperative Innovation Center for Sustainable Pig Production, Huazhong Agricultural University, Wuhan 430040, China; 80775@hnuahe.edu.cn (Y.L.); leimg@mail.hzau.edu.cn (M.L.); 3 College of Veterinary Medicine, Henan University of Animal Husbandry and Economy, Zhengzhou 450044, China

**Keywords:** *Escherichia coli*, carbapenem-resistance, colistin-resistance, *bla_NDM-1_*, *mcr-1*, plasmid, co-existence

## Abstract

The emergence of carbapenem-resistant and colistin-resistant *Enterobacteriaceae* represents a great risk for public health. In this study, the phenotypical and genetic characteristics of eight carbapenem-resistant and colistin-resistant isolates from pig farms in China were determined by the broth microdilution method and whole genome sequencing. Antimicrobial susceptibility testing showed that the eight carbapenem-resistant and colistin-resistant strains were resistant to three aminoglycosides, twelve β-lactams, one of the phenicols, one of the tetracyclines, and one of the fluoroquinolones tested, simultaneously. The prediction of acquired resistant genes using the whole genome sequences revealed the co-existence of *bla_NDM-1_* and *mcr-1* as well as the other genes that were responsible for the multidrug-resistant phenotypes. Bioinformatics analysis also showed that the carbapenem-resistant gene *bla_NDM-1_* was located on a putative IncFII-type plasmid, which also carried the other acquired resistant genes identified, including *fosA3*, *blaTEM-1B* and *rmtB*, while the colistin-resistant gene *mcr-1* was carried by a putative IncX4-type plasmid. Finally, we found that these resistant genes/plasmids were conjugative, and they could be co-conjugated, conferring resistance to multiple types of antibiotics, including the carbapenems and colistin, to the recipient *Escherichia coli* strains.

## 1. Introduction

*Escherichia coli* has become a great concern for global public health. On the one hand, the prevalence and outbreak of some intestinal pathogenic *E. coli* clones, particularly the well-known O157/ST11 and, more recently, the O104/ST678, has caused high levels of morbidity and mortality worldwide [1,2,3,4]; on the other hand, *E. coli* has had a great capacity to accumulate resistance genes, representing a natural reservoir of resistance genes, which may therefore contribute to the dissemination of antibiotic resistance and lead to treatment failures in both human and veterinary medicine [5]. It is of great importance to know how resistance genes are acquired and evaluate their capacity of dissemination between bacteria.

Recently, the emergence of the plasmid-mediated New Delhi metallo-β-lactamase-1 encoding gene *bla_NDM-1_* and/or the plasmid-mediated colistin-resistance gene *mcr-1* represents a great concern to global public health [6,7]. The plasmid-mediated *bla_NDM-1_* confers resistance to carbapenems [6], which is considered as the last resort for treating multidrug-resistant (MDR) *Enterobacteriaceae* [8], while *mcr-1* mediates the resistance to colistin [9], the key antibiotic used for treating carbapenem-resistant *Enterobacteriaceae* [8]. The emergence of carbapenems and colistin co-resistant *Enterobacteriaceae* means there will be little and/or no antibiotic available for the infections caused by such strains in most cases of infections caused by MDR *Enterobacteriaceae*. However, the co-existence of *bla_NDM_* and *mcr-1* in *Enterobacteriaceae* originated from both humans and animals has been increasingly reported worldwide [10,11,12,13]. These isolates are involved in the co-harboring of *mcr-1* and different members of *bla_NDM_* such as *bla_NDM-1_* [11], *bla_NDM-4_* [13], *bla_NDM-5_* [12], and *bla_NDM-9_* [10,14]. Of particularly concern is the recovery of an *E. coli* strain co-producing MCR-1 and NDM-9 from a patient with a catheter-associated urinary tract infection (UTI); the infection of such an *E. coli* led to the failure of antibiotic treatment and finally killed the patient [10]. Therefore, it is of great importance to monitor *Enterobacteriaceae* strains with co-resistance to carbapenems and colistin and understand how this resistance is accumulated. In this study, we report several carbapenem-resistant and colistin-resistant *E. coli* co-producing NDM-1 and MCR-1 from pig farms in China. 

## 2. Materials and Methods

### 2.1. Bacterial Strains and Antimicrobial Susceptibility Testing

Fecal and environmental swabs from 7 pig farms located in Hubei Province and Henan Province in China during June 2018 and June 2019 were collected for the isolation of carbapenem-resistant and colistin-resistant *E. coli*, using MacConkey agar containing 4 μg/ml of imipenem and 2 μg/ml of colistin. *E. coli* isolates were confirmed by PCR detection of the 16S rRNA gene and the seven house-keeping genes (*adk*, *fumC*, *gyrA*, *icd*, *mdh*, *purA*, and *recA*) of *E. coli* [15]. A total of 538 samples were collected and carbapenem-resistant and colistin-resistant *E. coli* were only detectable on samples from one farm in Henan province (sows ≥ 4000), where eight isolates were recovered from swabs of the floor and barrier of different pigsties (RDX007, RDX012), swabs of different food troughs (RDX020, RDX024), fecal samples of different pigs suffered from diarrhea (RDX033, RDX035, RDX100), and water troughs (RDX115).

The antimicrobial susceptibility of these eight isolates was determined by testing the minimal inhibitory concentration (MIC) values of the selected antibiotics on the bacteria using the broth microdilution method, according to the protocol recommended by the United Sates Clinical and Laboratory Standards Institute (CLSI M31-S1). A total of 28 types of antibiotics were assessed for the testing, namely, amikacin (AMK), gentamicin (GEN), tobramycin (TOB), imipenem (IPM), meropenem (MRP), ertapenem (ETP), colistin (CL), cefazolin (CFZ), cefuroxime (CFX), cefoxitin (FOX), ceftazidime (CAZ), ceftriaxone (CRO), cefepime (CPM), chloramphenicol (CHL), fosfomycin (FOS), nitrofurantoin (NIT), ciprofloxacin (CIP), levofloxacin (LVX), moxifloxacin (MXF), norfloxacin (NOR), minocycline (MIN), tetracycline (TET), aztreonam (AZM), tigecycline (TGC), amoxicillin/clavulanate (AMC), ampicillin/sulbactam (AMS), and piperacillin/tazobactam (PTZ), trimethoprim/sulfamethoxazole (SXT). These 28 types of antibiotics can be assigned to several classes: aminoglycosides (AMK, GEN, TOB), β-lactams (ETP, IPM, MRP, CFZ, CFX, FOX, CAZ, CRO, CPM, AMC, AMS, PTZ, AZM), Phenicols (CHL), tetracyclines (TET, MIN, TGC), fluoroquinolones (MXF, CIP, LVX, NOR), sulfonamides (SXT), fosfomycins (FOS), nitrofurantoins (NIT), and polymyxins (CL) (Sigma-Aldrich, USA). The thirteen β-lactam class of antibiotics can be re-assigned into four sub-classes: carbapenems (ETP, IPM, MRP), cephalosporins (CFZ, CFX, FOX, CAZ, CRO, CPM), β-lactam combination agents (AMC, AMS, PTZ), and monobactams (AZM). Results were interpreted using the CLSI breakpoints (CLSI-VET06, CLSI M100, 28th Edition). If a CLSI breakpoint is not available, a European Committee on Antimicrobial Susceptibility Testing (EUCAST) breakpoint is used for the interpretation. Each antibiotic was tested with three duplicates. *E. coli* ATCC 25922 was used as quality control.

### 2.2. Detection of the Carbapenem-Resistant and Colistin-Resistant Genes

PCR assays were initially performed to determine the presence and the location of the carbapenem-resistant gene *bla_NDM-1_* and colistin-resistant *mcr-1* with primers “F: GGTTTGGCGATCTGGTTTTC; R: CGGAATGGCTCATCACGATC” (for *bla_NDM-1_*, annealing temperature 55 °C, product size: 621 bp) [16] and “F: CGGTCAGTCCGTTTGTTC; R: CTTGGTCGGTCTGTAGGG” (for *mcr-1*, annealing temperature 55 °C, product size: 309 bp) [9], respectively. PCR reactions were performed in a 25 μl amplification mixture containing 2 μl of template DNA, 12.5 μl of 2×Taq Master Mix (Dye Plus, Vazyme Biotech Co., Ltd; Nanjing, China), 1 μl each of 10 μM forward and reverse primer, and 8.5 μl of nuclease-free water. The reaction was performed under the following cycling conditions: an initial denaturation at 95 °C for 10 min, followed by 35 cycles of denaturation at 94 °C for 45 s, annealing at 55 °C for 45 s, extension at 72 °C for 1 min, and a final extension at 72 °C for 10 min. PCR products were analyzed by electrophoresis on 1% agarose gel. Two rounds of PCR assays were performed. The first round of PCR assays was performed using the genomic DNA isolated from the strains as the template to determine the presence of the two resistant genes. An additional round of PCR assays was performed using the plasmids isolated from the strains to determine whether the detected resistant genes were located on the plasmids. 

### 2.3. DNA Extraction and Whole Genome Sequencing and Bioinformatic Analysis

Genomic DNA were extracted and purified using a TIANamp Bacteria DNA Kit (TIANGEN, Beijing, China). The quality and quantity of the DNA were evaluated by electrophoresis on a 1% agarose gel and using a NanoDrop2000 (Thermo Scientific, Waltham, USA). Whole genome sequencing was performed on an Illumina Hiseq Xten platform (Illumina Inc., San Diego, USA) at Guangdong Magigene Biotechnology Co. LTD (Guangzhou, China), using the pair-end 150 bp sequencing protocol. DNA libraries were constructed using a NEBNext Ultra^TM^ II DNA Library Prep Kit (New England BioLabs, Ipswich, USA). After sequencing, approximately 7,703,600~18,595,428 raw reads were yielded for the eight strains. Raw reads with low quality were filtered according to the following criteria: low quality base pairs at each terminal of the reads (Quality-Value < 20) were removed; reads with a short length (parameter setting at 50 bp), or > 15 bp overlap with Illumina TruSeq adapter sequences (parameter setting at 15 bp) were removed. Finally, approximately 7,363,540~17,856,944 clean reads (Q20% = 100, Q30% ≥ 95.59) were produced. High-quality reads were de novo assembled via SPAdes v3.9.0 [17] to generate contigs.

The assembled contigs were used for determining the genetic characteristics of the eight *E. coli* isolates. Serotypes and sequence types (ST) were determined by SerotypeFinder 2.0 [18] and Multi-Locus Sequence Typing (MLST) 2.0 [19], respectively. Acquired antimicrobial resistance genes and plasmids were determined using ResFinder 3.1 [20] and PlasmidFinder 2.0 [21], respectively. DNA identities between two sequences were calculated by ANI calculator [22]. A comparative genome analysis was performed and visualized using the BRIG package [23] and/or the EasyFig package [24]. Phylogenetic analysis was performed by the maximum likelihood method using the Tamura–Nei model [25] on MEGAX [26] with 1000 bootstrap iterations. The whole genome sequence of *E. coli* MG1655 (GenBank accession no. U00096.3) was downloaded from GenBank and used as the reference genome in the study.

The whole genome sequences of the eight *E. coli* isolates have been deposited into GenBank (BioProject accession no. PRJNA528830). GenBank accession numbers for each of the strains are: RXD007, SQQT00000000; RXD012: SQQW00000000; RXD020: SQQX00000000; RXD024: SQQY00000000; RXD033: SQQZ00000000; RXD035: SQRA00000000; RXD100: SQRC00000000; RXD115: SQRD00000000.

### 2.4. Plasmid Conjugation

Plasmid conjugation assays between the eight carbapenem-resistant and colistin-resistant *E. coli* (donor) and the rifampin resistant *E. coli* C600 (recipient) were performed on a nitrocellulose membrane, as described previously [27]. Briefly, mid-log phase donor and recipient strains (OD_600_ = 0.5~0.6) were mixed at a ratio of 1:3 (*v*/*v*). The bacterial mixture was then spotted on a nitrocellulose membrane which was pre-plated on the LB agar. After a 12 h of incubation at 37 °C, bacteria on the membrane were washed off using LB broth followed by being shaken at 37 °C for 4 h. Finally, the transconjugants were selected on LB agar plates laced with rifampin (1000 mgl^−1^) plus imipenem (20 mgl^−1^) plus colistin (2 mgl^−1^). Antimicrobial susceptibility of the transconjugants was determined using broth microdilution method as mentioned above. 

## 3. Results

### 3.1. Antimicrobial Susceptibility Profile of the Eight E. coli Isolates

**Figure 1:**
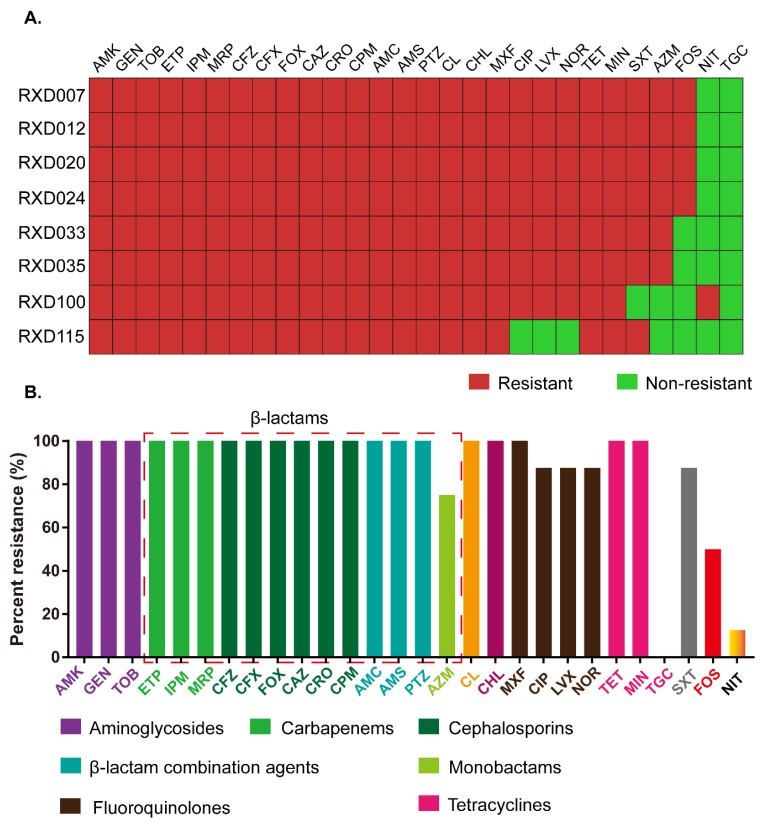
Antimicrobial susceptibility profile of the eight carbapenem-resistant and colistin-resistant *Escherichia coli* isolates. (**A**) Heatmap showing resistance and multidrug resistance phenotypes of each of the *E. coli* isolates; (**B**) Percent strains resistant to the tested antibiotics. AMK: amikacin; GEN: gentamicin; TOB: tobramycin; ETP: ertapenem; IPM: imipenem; MRP: meropenem; CFZ: cefazolin; CFX: cefuroxime; FOX: cefoxitin; CAZ: ceftazidime; CRO: ceftriaxone; CPM: cefepime; AMC: amoxicillin/clavulanate; AMS: ampicillin/sulbactam; PTZ: piperacillin/tazobactam; CL: colistin; CHL: chloramphenicol; MXF: moxifloxacin; CIP: ciprofloxacin; LVX: levofloxacin; NOR: norfloxacin; TET: tetracycline; MIN: minocycline; SXT: trimethoprim/sulfamethoxazole; AZM: aztreonam; FOS: Fosfomycin; NIT: nitrofurantoin; TGC: tigecycline.

Antimicrobial susceptibility testing showed that all (100%, 8/8) of the isolates tested herein were resistant to AMK, GEN, TOB, ETP, IPM, MRP, CFZ, CFX, FOX, CAZ, CRO, CPM, AMC, AMS, PTZ, CHL, MXF, TET, and CL (Figure 1). Many of them were also resistant to CIP (75.00%, 6/8), LVX (75.00%, 6/8), NOR (75.00%, 6/8), MIN (50.00%, 4/8), SXT (87.50%, 7/8), AZM (75.00%, 6/8) and FOS (50.00%, 4/8). Only one isolate was resistant to NIT (12.5%, 1/8). All of them were susceptible to TGC (100%, 8/8) (Figure 1A).

Regarding the different antibiotic classes, these eight carbapenem-resistant and colistin-resistant *E. coli* isolates were resistance to the three aminoglycosides tested (AMK, GEN, TOB), another nine β-lactams (CFZ, CFX, FOX, CAZ, CRO, CPM, AMC, AMS, PTZ) in addition to the three carbapenems tested, one of the phenicols tested (CHL), one of the tetracyclines tested (TET), one of the fluoroquinolones tested (MXF), simultaneously (Figure 1A). In addition, a higher proportion of these carbapenem-resistant and colistin-resistant *E. coli* strains showed resistance to another three fluoroquinolones tested (CIP, 75.00%; LVX, 75.00%; NOR, 75.00%), one of the β-lactams tested (AZM, 75.00%) and one of the sulfonamides tested (SXT, 87.50%). In total, 50% of the carbapenem-resistant and colistin-resistant *E. coli* strains were resistant to fosfomycin, and another one of the tetracyclines tested (MIN). A low proportion of the carbapenem-resistant and colistin-resistant *E. coli* strains displayed resistance to NIT (12.50%) and TGC (0.00%) (Figure 1B). Resistance to “AMK + GEN + TOB + ETP + IPM + MRP + CFZ + CFX + FOX + CAZ + CRO + CPM + AMC + AMS + PTZ + CL + CHL + MXF + TET” was the common characteristic for the eight carbapenem-resistant and colistin-resistant *E. coli* strains (Figure 1A).

### 3.2. Detection of the Carbapenem-Resistant Gene bla_NDM-1_ and Colistin-Resistant Gene mcr-1

**Figure 2:**
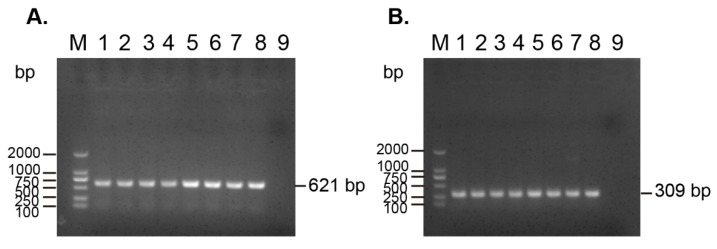
PCR detection of the carbapenem-resistant gene *bla_NDM-1_* and the colistin-resistant gene *mcr-1* from (**A**) the plasmids extracted from the eight carbapenem-resistant and (**B**) the colistin-resistant *E. coli* strains. M: DL 2000 DNA marker; 1–8: plasmid from RXD007, RXD012, RXD020, RXD024, RXD033, RXD035, RXD100, and RXD115; 9: ddH_2_O as negative control.

PCR assays revealed that the eight carbapenem-resistant and colistin-resistant *E. coli* strains contained the carbapenem-resistant gene *bla_NDM-1_* and colistin-resistant gene *mcr-1*, simultaneously. In addition, PCR assays using plasmids extracted from the eight strains as template showed positive results for the detection of the two genes, suggesting that the two genes were located on plasmids (Figure 2). 

### 3.3. Acquired Antibiotic Resistance Genes in the Eight Carbapenem-Resistant and Colistin-Resistant E. coli

**Figure 3:**
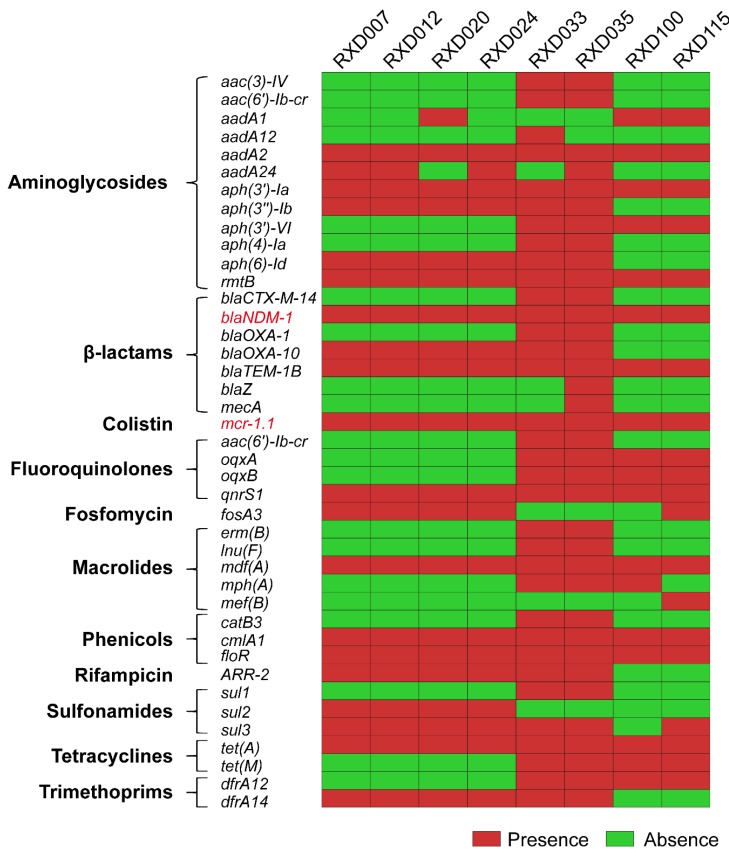
Heatmap showing acquired resistant genes identified in each of the eight carbapenem-resistant and colistin-resistant *E. coli* isolates.

The whole genome sequencing strategy revealed that each of the eight carbapenem-resistant and colistin-resistant *E. coli* isolates possessed a chromosome of approximately 5.0 Mb in size and several plasmid contigs. The prediction of acquired resistance genes using whole genome sequences identified the presence of *bla_NDM-1_* (*n* = 8), *bla_TEM-1B_* (*n* = 8), *bla_OXA-10_* (*n* = 6), *bla_CTX-M-14_* (*n* = 2), *bla_OXA-1_* (*n* = 2), *bla_Z_* (*n* = 1), and *mecA* (*n* = 1), which account for the resistance to carbapenem and the other β-lactam antibiotics (Figure 2). These strains also carried *aph(3′)-Ia* (*n* = 8), *aadA2* (*n* = 8) and *rmtB* (*n* = 8), accounting for the resistance to aminoglycosides; *floR* (*n* = 8) and *cmlA1* (*n* = 8), accounting for the resistance to phenicols; *tet(A)* (*n* = 8) and *tet(M)* (*n* = 4), accounting for the resistance to tetracyclines; *qnrS1* (*n* = 8), *oqxA* (*n* = 4), *oqxB* (*n* = 4), and *aac(6′)-Ib-cr* (*n* = 2), accounting for the resistance to fluoroquinolones; *sul3* (*n* = 7), *sul2* (*n* = 4) and *sul1* (*n* = 2), accounting for the resistance to sulfonamides; *fosA3* (*n* = 8), which accounts for osfomycin resistance, and *mcr-1.1* (*n* = 8), which accounts for colistin resistance (Figure 3).

### 3.4. Genetic Characteristics

**Figure 4:**
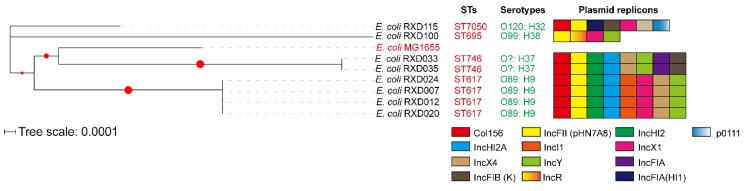
Phylogenetic analysis of the eight carbapenem-resistant and colistin-resistant *E. coli* isolates. Sequence types, O-serotypes, H-serotypes, and plasmid replicons are also shown. The evolutionary history was inferred by using the Maximum Likelihood method and Tamura–Nei model. The tree with the highest log likelihood (−13321.25) is shown. The percentage of trees in which the associated taxa clustered together is shown next to the branches. Initial tree(s) for the heuristic search were obtained automatically by applying Neighbor-Join and BioNJ algorithms to a matrix of pairwise distances estimated using the Maximum Composite Likelihood (MCL) approach, and then selecting the topology with superior log likelihood value. The tree is drawn to scale, with branch lengths measured in the number of substitutions per site. This analysis involved nine nucleotide sequences. There was a total of 9093 positions in the final dataset. Evolutionary analyses were conducted by using MEGA X. The circles denote bootstrap values within the range of 0.19–1.000.

Multilocus sequence typing (MLST) identified four kinds of sequence types: ST617 (*n* = 4), ST746 (*n* = 2), ST695 (*n* = 1), and ST7050 (*n* = 1) (Figure 4). Three types of O-serotypes and four types of H-serotypes were determined: O89: H9 (*n* = 3), O89: H10 (*n* = 1), O99: H38 (*n* = 1), and O120: H32 (*n* = 1) (Figure 3). The prediction of plasmid determined four to eight replicons in each of the genomes. Among these replicons, a predicted replicon of IncFII-type plasmid pHN7A8 (GenBank accession: JN232517) was carried by all of the carbapenem-resistant and colistin-resistant *E. coli* strains (Figure 4). 

### 3.5. Plasmid Determination

**Figure 5:**
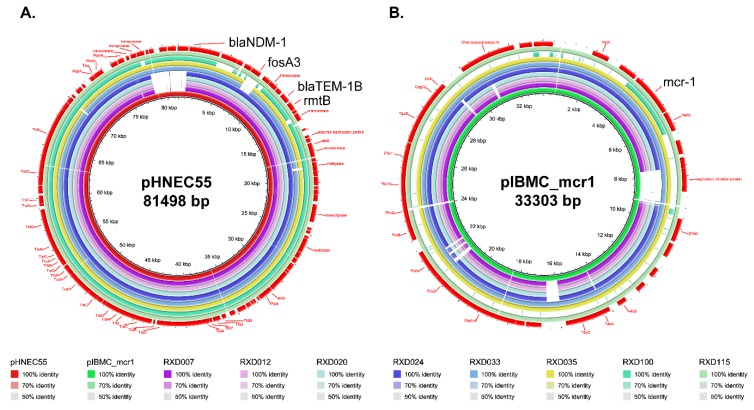
Comparison of plasmid pHNEC55 (panel A) and pIBMC_mcr1 (panel B) with the genomes of RXD007, RXD012, RXD020, RXD024, RXD033, RXD035, RXD100, and RXD115, displayed as the outer rings inside to outside, respectively. The outmost rings (red) show the functional genes including the resistance genes as well as the structural genes carried by pHNEC55 (panel A) and pIBMC_mcr1 (panel B).

Because the results of the PCR assays revealed that the *bla_NDM-1_* gene and the *mcr-1* gene were located on plasmids (Figure 2), we used the whole genome sequences to analyze the putative *bla_NDM-1_*-carring and/or *mcr-1*-carrying plasmids that were harbored by the eight carbapenem-resistant and colistin-resistant *E. coli* isolates. Bioinformatical analysis revealed the *bla_NDM-1_* gene was located on an IncFII-type plasmid, while the *mcr-1.1* gene carried by RXD007, RXD012, RXD020, RXD024, RXD033, RXD035, and RXD115 was located on an IncX4-type plasmid. However, we did not find the putative *mcr-1*-carrying plasmid in RXD100 showing any homologous to any plasmids with DNA sequences available in GenBank. The IncFII-type plasmid carrying the *bla_NDM-1_* gene was homologous to pHNEC55 (GenBank accession: KT879914), and this pHNEC55-like plasmid also carried *fosA3*, *blaTEM-1B* and *rmtB* (Figure 5A). An IncX4-type plasmid carrying *mcr-1.1* presence in RXD007, RXD012, RXD020, RXD024, RXD033, RXD035, and RXD115 was homologous to pIBMC_mcr1 (GenBank accession: MF449287) (Figure 5B).

### 3.6. Plasmid Conjugation

To assess the transferability of the plasmids and the resistance genes, conjugation experiments between the eight were performed with selective plates laced with rifampin (1000 mgl^−1^) plus imipenem (20 mgl^−1^) plus colistin (2 mgl^−1^). Transconjugants incubated on selective agars were selected for antimicrobial susceptibility testing. The result showed that the plasmids in seven of the eight carbapenem-resistant and colistin-resistant *E. coli* transferred into the recipient strain and after conjugation, the resistance phenotypes of the recipient *E. coli* (C600) changed remarkably (Table 1).

## 4. Discussion

Carbapenem and colistin are the last-resort antibiotics used for treating multidrug-resistant Gram-negative pathogens [8]. It is of great public health significance to monitor the carbapenem-resistant and colistin-resistant bacteria and investigate the mechanisms for the acquisition of the resistant phenotypes. In this study, we isolated eight carbapenem-resistant and colistin-resistant *E. coli* from a pig farm in Henan Province, China. Antimicrobial susceptibility testing revealed the eight carbapenem-resistant and colistin-resistant isolates were also resistant to aminoglycosides, cephalosporins, β-lactam combination agents, phenicols (CHL), tetracyclines, fluoroquinolones, and sulfonamides (SXT) (Figure 1 and Figure 2). Since most of these phenotypes in *E. coli* were conferred by resistance genes mostly through horizontal gene transfer [5], the presence of those strains in pigs/farms may represent a real public health concern: (1) these MDR isolates might transmit between pigs/environment and other animal species including humans through numerous pathways such as via direct/indirect contact and/or via food-chain, which might therefore lead to treatment failures in both human and veterinary medicine; (2) such strains might also act as a major reservoir of resistance genes, which might contribute to the spread of antimicrobial resistance.

Corresponding to the resistant phenotypes determined, prediction using the whole genome sequences identified many types of acquired genes conferring resistance to β-lactams (including cephalosporins and carbapenems), aminoglycosides, fluoroquinolones, polymyxins, tetracyclines, phenicols, sulfonamides, trimethoprim, and fosfomycin (Figure 3). Of particularly note is the *bla_NDM-1_* gene, which encodes the New Delhi metallo-β lactamase 1 (*bla_NDM-1_*) and confers the resistance to carbapenems [28,29], and the *mcr-1* gene, which encodes the phosphoethanolamine-lipid A transferase and confers the resistance to colistin [9]. Both of these two genes have substantial importance worldwide in terms of resistance [7,29,30,31] and they were identified in the eight carbapenem-resistant and colistin-resistant *E. coli* strains (Figure 2 and Figure 3), suggesting that the co-existence of *bla_NDM-1_* and *mcr-1* mediates the resistance phenotypes to carbapenems and colistin.

Three of the eight carbapenem-resistant and colistin-resistant *E. coli* were isolated from fecal samples of different diarrheal pigs. Two of them, designated RDX033 and RDX035, were found to be ST746 (Figure 4). It is worth noting that MCR-1-carrying, extended-spectrum β-lactamase (ESBL)-producing *E. coli* ST746 has been recovered from community-acquired urinary tract infection [32]. The human sourced *E. coli* ST746 contained plasmid-carrying *mcr-1* as well as two kinds of β-lactam-resistant genes *bla*_CTX-M-14_ and *bla*_TEM-1B_ but not *bla*_NDM-1_ gene [32]. However, the two ST746 strains recovered from diarrheal pigs carried both *mcr-1* and *bla*_NDM-1_ in addition to *bla*_CTX-M-14_ and *bla*_TEM-1B_ (Figure 3), and they displayed a broader spectrum of resistance compared with the human sourced ST746. The identification of such strains in pigs might represent a potential risk on public health. Another carbapenem-resistant and colistin-resistant *E. coli* recovered from different diarrheal pigs was RXD100, which was identified as ST695. While this isolate showed resistance to most of the antibiotics tested, it was not as resistant as the ST746 strains RDX033 and RDX035 (Figure 1). Fewer numbers of resistant genes carried by RXD100 compared to RDX033 and RDX035 might explain the difference (Figure 3).

It has been widely reported that plasmids play an important role in the dissemination of *bla_NDM-1_* and *mcr-1* [5,9,29,31]. Consistently, our PCR results using the plasmids extracted as templates from the eight strains revealed that both of the genes were located on plasmids (Figure 2). Plasmid prediction using whole genome sequences identified several putative types of plasmids harbored by the eight strains (Figure 4). Among these putative types of plasmids, IncHI2A, IncFIB(K), IncFII, IncFI1, IncR, IncHI2, and IncFIA plasmids carrying *bla_NDM-1_* have been reported [33,34,35,36,37], while IncX4, IncFIB(K), IncFII, IncFI1, IncY, IncHI2, IncX1, and IncFIA plasmids carrying *mcr-1* have been reported [38,39,40,41,42]. Although more accurate and reliable technologies such as the third-generation sequencing technologies are necessary for determining the accurate type and sequence of the plasmids, bioinformatical analysis using whole genome sequences revealed that an IncFII-type plasmid homologous to pHNEC55 (GenBank accession: KT879914) likely carried the *bla_NDM-1_* gene, and an IncX4-type plasmid homologous to pIBMC_mcr1 (GenBank accession: MF449287) likely carried the *mcr-1* gene, as the backbones of both plasmids were found in the genomes of the eight strains (Figure 5). Interestingly, the IncFII plasmid pHNEC55 carrying *bla_NDM-1_* was also identified in a carbapenem-resistant *E. coli* strain isolated from pigs in Henan Province [43]. The multidrug resistance-encoding mobile elements of this plasmid contains several other resistant genes in addition to *bla_NDM-1_*, including *mphA*, which confers resistance to macrolides; *fosA3*, which confers resistance to Fosfomycin; *rmtB*, which confers resistance to aminoglycosides; and *bla*_TEM_, which confers resistance to β-lactams [43]. These genes were also carried by the eight carbapenem-resistant and colistin-resistant bacteria and they were also likely to be carried by the *bla_NDM-1_*-carrying plasmids (Figure 5A). In the next step, we intend to use more accurate and reliable technologies such as third-generation sequencing or a combination of second-generation with third-generation sequencing to determine the complete genome sequences and the accurate structures of the MDR plasmids in the eight *E. coli* strains.

Plasmid conjugation assays revealed that the MDR plasmids harbored by the eight carbapenem-resistant and colistin-resistant bacteria were transferrable, and it seems that these MDR plasmids are capable of co-conjugation at appropriate conditions, as the conjugation of the plasmids conferred both carbapenem and colistin resistance to the recipient bacteria simultaneously (Table 1). The same results have also been found in other articles [14]. In addition to carbapenems and colistin, the conjugation of these plasmids was also able to disseminate resistance to many types of antibiotics tested, including aminoglycosides, cephalosporins, β-lactam combination agents, phenicol, tetracyclines, fluoroquinolones, and sulfonamides (Table 1). Many of these antibiotics are commonly used antibiotics for bacterial infections in both human and veterinary medicine [5], and many are classified as critically important antimicrobials by the WHO and their usage should be severely restricted (https://www.who.int/foodsafety/publications/antimicrobials-fifth/en/). Therefore, the presence of these conjugative MDR plasmids might lead to the spread of the MDR plasmids and treatment failures in both human and veterinary medicine. More active actions should be taken to monitor the prevalence of such plasmids as well as their bacterial hosts. In the next step, we will evaluate the conjugative capacity and efficacy of each of the resistant plasmids after their complete genome sequences are determined by third-generation sequencing technologies. 

## 5. Conclusions

In conclusion, a total of eight carbapenem-resistant and colistin-resistant *E. coli* from pigs/farms in Central China were characterized in the present study. The carbapenem-resistant and colistin-resistant *E. coli* isolates were commonly resistant to aminoglycosides, cephalosporins, β-lactam combination agents, phenicol, tetracyclines, fluoroquinolones, and sulfonamides. A coexistence of a putative IncFII-type plasmid homologous to pHNEC55 and an IncX4-type plasmid homologous to pIBMC_mcr1 is likely to be responsible for the dissemination of the carbapenem-resistant gene *bla_NDM-1_* and colistin-resistant gene *mcr-1*, and both genes/plasmids are conjugative and could be co-conjugatively transferred. In the next study, we intend to use more accurate and reliable technologies like third-generation sequencing to determine the complete genome sequences and the accurate structures of these MDR plasmids.

## Figures and Tables

**Table 1:** Phenotypic characteristics of the transconjugants of the eight carbapenem-resistant and colistin-resistant *E. coli.*

Antibiotics tested	Transconjugants selected by rifampin plus imipenem plus colistin							E. coli C600
	Minimum inhibitory concentration (μg/ml)							
	Cir007 ^1^	Cir012	Cir020	Cir033	Cir035	Cir100	Cir115	
Amikacin	> 32 (R^1^)	> 32 (R)	> 32 (R)	> 32 (R)	> 32 (R)	> 32 (R)	> 32 (R)	≤ 8 (S)
Gentamicin	> 8 (R)	> 8 (R)	> 8 (R)	> 8 (R)	> 8 (R)	> 8 (R)	> 8 (R)	≤ 2 (S)
Tobramycin	> 8 (R)	> 8 (R)	> 8 (R)	> 8 (R)	> 8 (R)	> 8 (R)	> 8 (R)	≤ 2 (S)
Ertapenem	> 2 (R)	> 2 (R)	> 2 (R)	> 2 (R)	> 2 (R)	> 2 (R)	2 (R)	≤ 0.25 (S)
Imipenem	> 8 (R)	> 8 (R)	> 8 (R)	> 8 (R)	> 8 (R)	1 (S)	8 (R)	0.5 (S)
Meropenem	> 8 (R)	> 8 (R)	> 8 (R)	> 8 (R)	> 8 (R)	1 (S)	4 (R)	≤ 0.13 (S)
Cefazolin	> 16 (R)	> 16 (R)	> 16 (R)	> 16 (R)	> 16 (R)	> 16 (R)	> 16 (R)	4 (S)
Cefuroxime	> 16 (R)	> 16 (R)	> 16 (R)	> 16 (R)	> 16 (R)	> 16 (R)	> 16 (R)	16 (R)
Cefoxitin	> 16 (R)	> 16 (R)	> 16 (R)	> 16 (R)	> 16 (R)	> 16 (R)	> 16 (R)	8 (S)
Ceftazidime	> 32 (R)	> 32 (R)	> 32 (R)	> 32 (R)	> 32 (R)	> 32 (R)	> 32 (R)	≤ 1 (S)
Ceftriaxone	> 32 (R)	> 32 (R)	> 32 (R)	> 32 (R)	> 32 (R)	> 32 (R)	> 32 (R)	≤ 1 (S)
Cefepime	> 16 (R)	> 16 (R)	> 16 (R)	16 (R)	> 16 (R)	16 (R)	8 (R)	≤ 1 (S)
Amoxicillin/clavulanate	> 32/16 (R)	> 32/16 (R)	> 32/16 (R)	> 32/16 (R)	> 32/16 (R)	32/16 (R)	32/16 (R)	≤ 8/4 (S)
Ampicillin/sulbactam	>16/8 (R)	>16/8 (R)	>16/8 (R)	>16/8 (R)	> 16/8 (R)	> 16/8 (R)	>16/8 (R)	8/4 (S)
Piperacillin/tazobactam	>64/4 (R)	>64/4 (R)	>64/4 (R)	>64/4 (R)	>64/4 (R)	>64/4 (R)	>64/4 (R)	≤ 4/4 (S)
Colistin	> 4 (R)	> 4 (R)	4 (R)	4 (R)	4 (R)	> 4 (R)	> 4 (R)	≤ 1 (S)
Chloramphenicol	> 16 (R)	> 16 (R)	> 16 (R)	≤ 4 (S)	≤ 4 (S)	> 16 (R)	≤ 4 (S)	≤ 4 (S)
Moxifloxacin	> 2 (R)	> 2 (R)	> 2 (R)	≤ 0.5 (S)	≤ 0.5 (S)	> 2 (R)	≤ 0.5 (S)	≤ 0.5 (S)
Ciprofloxacin	> 4 (R)	> 4 (R)	> 4 (R)	≤ 0.5 (S)	≤ 0.5 (S)	2 (I)	≤ 0.5 (S)	≤ 0.5 (S)
Levofloxacin	> 8 (R)	> 8 (R)	> 8 (R)	≤ 1 (S)	≤ 1 (S)	2 (S)	≤ 1 (S)	≤ 1 (S)
Norfloxacin	> 8 (R)	> 8 (R)	> 8 (R)	≤ 2 (S)	≤ 2 (S)	4 (S)	≤ 2 (S)	≤ 2 (S)
Tetracycline	> 8 (R)	> 8 (R)	> 8 (R)	≤ 2 (S)	≤ 2 (S)	> 8 (R)	≤ 2 (S)	≤ 2 (S)
Minocycline	4 (S)	4 (S)	4 (S)	≤ 1 (S)	2 (S)	16 (R)	2 (S)	≤ 1 (S)
Trimethoprim/sulfamethoxazole	> 4/76 (R)	> 4/76 (R)	> 4/76 (R)	≤1/19 (S)	≤ 1/19 (S)	≤ 1/19 (S)	≤1/19 (S)	≤ 1/19 (S)
Aztreonam	≤ 2 (S)	≤ 2 (S)	≤ 2 (S)	≤ 2 (S)	≤ 2 (S)	≤ 2 (S)	≤ 2 (S)	≤ 2 (S)
Fosfomycin	> 128 (R)	> 128 (R)	> 128 (R)	≤ 16 (S)	≤ 16 (S)	≤ 16 (S)	> 128 (R)	≤ 16 (S)
Nitrofurantoin	≤ 16 (S)	32 (S)	≤ 16 (S)	≤ 16 (S)	≤ 16 (S)	> 64 (R)	≤ 16 (S)	≤ 16 (S)
Tigecycline	≤ 1 (S)	≤ 1 (S)	≤ 1 (S)	≤ 1 (S)	≤ 1 (S)	≤ 1 (S)	≤ 1 (S)	≤ 1 (S)

^1^ Note: Cir007, Cir012, Cir020, Cir033, Cir035, Cir100, and Cir115 represent the transconjugant of RXD007, RXD012, RXD020, RXD033, RXD035, RXD100, and RXD115, respectively. R: Resistant; S: susceptible; I: intermediately resistant.
